# Suicide-Related Emergency Department Visits Before and During the COVID-19 Pandemic in the United States

**DOI:** 10.7759/cureus.68205

**Published:** 2024-08-30

**Authors:** Melissa N Litenski, Yulia Shtanko, Aoife B O'Reardon, Grettel Castro, Daniel Castellanos, Marcia Varella

**Affiliations:** 1 Medical School Department of Psychiatry and Behavioral Health, Herbert Wertheim College of Medicine, Miami, USA; 2 Medical School Department of General Surgery, Herbert Wertheim College of Medicine, Miami, USA; 3 Department of Medical and Population Health Sciences Research, Herbert Wertheim College of Medicine, Miami, USA; 4 Department of Psychiatry, Creighton University School of Medicine, Phoenix, USA

**Keywords:** public mental health, intentional injury, suicide attempt, emergency department visits, coronavirus pandemic, covid-19

## Abstract

Background and aim

The impact of COVID-19 on suicide rates is a significant concern, given the widely recognized psychological effects that the pandemic has had on mental health. Overall, suicide trends remained relatively stable. Yet, specific age groups, races, and genders experienced an increase in suicide rates. A better understanding of suicide trends over time is critical to identifying and addressing mental health crises exacerbated by the pandemic. This study aimed to study whether the years preceding and during the pandemic were associated with an increase in emergency department (ED) visits in the United States for suicide or intentional injury.

Methodology

Secondary analyses of data from the National Hospital and Ambulatory Medical Care Survey (2018-2021) were conducted. The frequency of ED visits due to intentional injury or suicide was compared in 2018-2019 (pre-COVID-19 pandemic onset) to those of 2020-2021 (during-COVID-19 onset). Logistic regression was used to estimate odd ratios (ORs) and corresponding 95% confidence intervals. Patient’s race, sex, age, and regional differences were assessed as covariates.

Results

There were 27,516 and 22,247 visits assessed in the pre- and during-COVID-19 periods, respectively. In total, 1,375 visits were due to intentional injury/suicide. No differences were found comparing the proportion of visits due to intentional injuries/suicide pre- and during-COVID-19 periods (2.6% in both) The adjusted OR (aOR) comparing pre- versus during-COVID-19 for emergency room visits due to intentional injury/suicide was not significantly different from 1 (aOR = 0.98, 95% CI 0.84-1.15). The odds of suicide/intentional injury were 53% higher in males (aOR = 1.53, 95%CI 1.30-1.81), in those with ages 18-44 years (aOR = 7.24, 95% CI 4.92-10.67) and 45-64 years (aOR = 3.55, 95% CI 2.31-5.47) compared to those 65 years or older, and in non-Hispanic Black individuals compared to non-Hispanic White individuals (aOR = 1.29, 95% CI 1.05-1.58).

Conclusions

Using a national sample of ED visits, we found no association between the pre- and COVID-19 pandemic periods and the proportion of visits due to intentional injury/suicide. However, the study's proportional prevalence design limits its ability to estimate actual risk, requiring a cautious interpretation of the findings. Despite these limitations, the observed increased odds of suicide or intentional injury in specific subgroups underscore the need for targeted interventions. Further research is crucial to assess the long-term impacts of COVID-19.

## Introduction

The COVID-19 pandemic brought about increased stress and isolation due to restrictions, work-from-home protocols, and shifts in family dynamics [1]. Concurrently, there was a surge in social media campaigns aimed at promoting mental health and reducing stress to foster interconnectedness [2]. These efforts were vital in mitigating feelings of isolation and stress, known contributors to suicide. In the early months of the pandemic, suicide deaths remained largely stable or even decreased in developed nations [3,4]. However, as 2020 progressed, these initial patterns began to shift. 

China, as the initial epicenter of the COVID-19 outbreak, took early initiatives to investigate the pandemic's impact on mental health. A survey of 1,210 participants in China, conducted through an online survey on a dedicated university website, revealed that more than 650 (>50%) reported severe psychological impacts due to the COVID-19 pandemic [5]. Another study assessed college students for post-traumatic stress disorder (PTSD) symptoms using the Impact of Event Scale-Revised scale during the early months of the pandemic. Results indicated that out of 9,274 participants, 1,211 (13.1%) of students exhibited PTSD symptoms [6], in contrast to another study conducted one month after the outbreak of 589 participants, in which 27 individuals (4.6%) reported PTSD symptoms [7]. 

Beyond China, several other countries also experienced deleterious impacts of COVID-19. Japan, saw an increase in suicide rates during the pandemic, particularly between August and November 2020 [8]. Emergency department (ED) admissions from December 30, 2018, to October 10, 2020, further indicated higher median visit counts for suicide attempts, overdoses, and incidents of violence compared to the same period in the previous year [9,10]. In South Korea, a retrospective cohort study in a university hospital from January 2018 to December 2020 observed an increase in suicide attempts among women, adults, and the elderly, with a particular rise in impulsive suicides involving alcohol [1]. 

Contrary to this, several other nations, including Canada, witnessed a decline in suicide rates. A cross-sectional study delving into national suicide rates during the pandemic and evaluating the impact of social service programs reported a decrease in suicide rates during the pandemic [11], potentially influenced by heightened government interventions. While certain countries reported an overall decrease in suicide rates, specific subsets of their populations exhibited divergent trends. An ecological study in Brazil of 10,409 individuals reported a 19% reduction in suicides among individuals aged 10-29 during the pandemic, from 2020 to 2021 [12]. However, the same study identified an increase in suicide rates among selected subgroups, particularly older individuals residing in areas marked by socioeconomic inequity [12]. Similarly, in Taiwan, an interrupted time-series analysis revealed an overall decrease in suicide rates after the pandemic, propelled by fewer suicide deaths among middle-aged individuals. Nevertheless, there was a concurrent increase in suicide rates among younger and older age groups [13]. 

A systematic review and meta-analysis encompassing data from thirteen studies up to December 2022 examining pre- and peri-pandemic rates of suicidal ideation, suicide attempts, and suicide mortality [14]. This work demonstrated that within the United States, there was an increase in both suicidal ideation and suicide attempts during the COVID-19 pandemic, despite the overall suicide rate remaining stable [14]. 

The overall discrepancies across studies and countries regarding the impact of the COVID-19 pandemic on suicide suggested the need to further explore the pandemic's impact on mental health. The present study aims to expand on current literature by analyzing ED visits for incidents of intentional injury or suicide, offering a focused examination of trends in visits for suicide attempts and/or self-harm during the pandemic compared to pre-pandemic periods. This article was previously presented as an oral presentation at the Herbert Wertheim College of Medicine Tenth Annual Research Symposium on April 19, 2024, and as a poster at the University of Miami 2024 National Global Health and Surgery Conference on April 27, 2024. 

## Materials and methods

The study was a secondary analysis of data from the National Hospital and Ambulatory Health Care Survey (NHAMCS) from 2018 to 2021. The NHAMCS collects data including, but not limited to, the reason for the visit, age, sex, and race of patients visiting the ED of participating hospitals across the United States. Participating hospitals complete a survey regarding their hospital system, and this data is collected and analyzed, and the responses are weighted. Importantly, this study relied on the abstraction of data from hospital visit records, not administrative billing records. The NHAMCS is a national survey based on a complex random national sample of ED visits in noninstitutional general and short-stay hospitals. Information is abstracted from medical records by specially trained interviewers to ensure accuracy and consistency in data collection.

The data set was categorized based on two independent periods: *pre-COVID-19* (years 2018-2019) and *during-COVID-19* (years 2020-2021). To determine if a visit was related to mental health or suicide, the International Classification of Diseases, 10th Revision, Clinical Modification (ICD-10-CM) diagnosis codes F01 to F99 were used. After identifying relevant visits through these codes, the visit had to be marked *YES* under categories such as "related to injury/trauma, overdose/poisoning, or adverse effect of medical/surgical treatment," and also marked *YES* under "intentional" to be considered as a suicide/mental health-related visit. The covariates considered included sex (male, female), race (non-Hispanic White, non-Hispanic Black, Hispanic, and non-Hispanic others), age groups (18-44 years old, 45-64 years old, and >65 years old), and geographical location of residency within the United States (Northeast, Midwest, Southern, and Western areas). 

Descriptive exploratory analyses were first conducted to characterize the sample. Bivariate analyses were then performed to examine the distribution of characteristics based on calendar year categories (pre-pandemic and during pandemic onset), as well as the counts of suicide attempt-related visits by year and selected characteristics. Finally, logistic regression analysis was used to assess the association between calendar year and ED visits for intentional suicide attempts, with odds ratios (ORs) and 95% confidence intervals estimated.

## Results

We assessed data from 54,996 individuals, 18 years and older, who visited the ED in 2018-2021. We excluded data from individuals with missing information on intentional injury or suicide (n=5,233). Overall, 49,763 suicide-related visits had complete information on key variables and were included for analysis. Table 1, displays the baseline demographic characteristics of the sample used from NAMCS/NHAMCS pre-COVID-19 compared to during COVID-19 periods. Females had slightly higher proportions pre-COVID-19 (*n* = 15,363, 57%) than during-COVID-19 (*n* = 11,939, 55%). The Northeast and Midwest regions also showed slightly higher proportions during COVID-19 compared to pre-COVID-19 (Northeast: *n* = 5,896, 16.30%, pre-COVID-19 to *n* = 4,605, 17.20%, during COVID-19; Midwest: *n* = 6,212, 19.30%, pre-COVID-19 to *n* = 5,047, 20.50%, during COVID-19). In contrast, there were increases in the proportion of visits from the South (*n* = 9,908, 36.70%, during-COVID-19 compared to *n* = 6,868, 42.60%, pre-COVID-19) and the West (*n* = 5,500, 21.80%, pre-COVID-19 to *n* = 5,727, 25.60%, during COVID-19).

**Table 1:** Baseline characteristics of the NAMCS/NHAMCS dataset sample. Analysis was performed by using a chi-square analysis, in which the chi-square value is *P *< 0.05.  The *N* values are presented as unweighted to reflect the actual number of observations for each category in the dataset. In contrast, the percentages are weighted to account for the complex sampling design. This design involves varying probabilities of selection for different respondents, and each respondent is assigned a weight based on the number of people they represent in the US population. NHAHCS, National Hospital and Ambulatory Health Care Survey

Characteristics	Time					Statistical test
	Pre-COVID-19 (2018-2019)		During-COVID-19 (2020-2021)		*P*-value	
	Unweighted *N*	Weighted %	Unweighted *N*	Weighted %		
Sex					0.009	Chi-square analysis (*P *< 0.05)
Female	15,363	57.00	11,939	55.10		
Male	12,153	43.00	10,308	44.90		
Age					0.144	Chi-square analysis (*P *< 0.05)
18-44	13,592	48.30	10,696	47.40		
45-64	8,162	29.70	6,572	29.30		
65+	5,762	22.10	4,979	23.30		
Region					0.027	Chi-square analysis (*P* < 0.05)
Northeast	5,896	16.30	4,605	17.20		
Midwest	6,212	19.30	5,047	20.50		
South	9,908	42.60	6,868	36.70		
West	5,500	21.80	5,727	25.60		
Race					0.292	Chi-square analysis (*P* < 0.05)
Non-Hispanic White	15,635	58.30	12,934	60.86		
Non-Hispanic Black	6,907	24.40	5,248	21.80		
Hispanic	3,915	13.80	3,125	13.60		
Non-Hispanic Others	1,059	3.42	940	3.74		

Overall a total of 595 (2.6%) visits to the emergency department in the study period were due to suicide/intentional injury. There was no significant difference in the frequency of those who presented to the emergency department with suicidal behaviors pre-COVID-19 compared to during-COVID-19, aOR = 0.98, with *P*-value = 0.847 (Table 2). A higher percentage of males (*n* = 748, 3.12%) reported intent of injury being suicide during ED visits compared to females (3.1% vs. 2.6%, *n* = 627, *P* < 0.001). Individuals aged 18-44 years had the highest proportion of intentional injury-related ED visits (*n *= 992, 3.91%), and those aged 65+ had a lower frequency (*n *= 99, 0.55%). Non-Hispanic Black individuals had a higher proportion of suicide-related ED visits (n = 434, 3.33%) compared to Hispanic individuals (*n* = 213, 2.76%), non-Hispanic White individuals (*n* = 669, 2.27%), and non-Hispanic Others (*n* = 1,375, 2.25%) (*P* < 0.001). Finally, no differences in suicide/intentional injury were significant among the regions.

**Table 2:** Proportional prevalence of suicide/intent of injury according to selected characteristics, including time, sex, age, race, and region. Analysis was performed by using chi-square analysis, *P *< 0.05.

Characteristics		Reason for ED visit				Statistical test
	Other reason/diagnosis		Suicide/Intentional injury		*P*-value	
	Unweighted *N*	Weighted %	Unweighted *N*	Weighted %		
Time					0.847	Chi-square analysis (*P *< 0.05)
Pre-COVID-19 (2018-2019)	26,736	97.40	780	2.60		
During-COVID-19(2020-2021)	21,652	97.40	595	2.60		
Sex					<0.001	Chi-square analysis (*P* < 0.05)
Female	26,675	97.80	627	2.60		
Male	21,713	96.70	748	3.12		
Age					<0.001	Chi-square analysis (*P *< 0.05)
18-44	23,296	96.10	992	3.91		
45-64	14,410	98.00	324	1.99		
65+	10,682	99.40	99	0.55		
Region					0.091	Chi-square analysis (*P *< 0.05)
Northeast	10,194	97.30	307	2.73		
Midwest	10,946	93.00	313	2.98		
South	16,363	93.80	413	2.25		
West	10,885	97.30	342	2.68		
Race					<0.001	Chi-square analysis (*P *< 0.05)
Non-Hispanic White	27,900	97.70	669	2.27		
Non-Hispanic Black	11,721	96.70	434	3.33		
Hispanic	6,827	97.20	213	2.76		
Non-Hispanic Others	1,940	97.40	1375	2.25		

The OR for the association between the pre- and post-COVID-19 periods and the proportional prevalence of suicide attempt ER visits was not statistically different from 1, indicating no evidence of an association (OR = 0.98, 95% CI 0.84-1.16) (Table 3). After adjusting for participant’s sex, age, race, and region, the OR for suicide-related ED visits when comparing pre-COVID-19 years to during-COVID-19 years was 0.98 (95% CI 0.84-1.15), indicating no evidence of a difference in the likelihood of suicide-related ED visits between the two periods. Additionally, males were 58% more likely to visit the ED for intentional injury compared to women (OR = 1.53, 95% CI 1.30-1.81; *P *≤ 0.001). When comparing younger (18-44 years) to older (65+) age groups, the younger population had a seven-fold increase in ED visits for suicidal behaviors compared to the older population (OR = 7.24, 95% CI 4.92-10.67; *P *≤ 0.001). A comparison of adults 44-65 years old to those 65 and older revealed a 3.5-fold increase in the rate of ED visits (OR = 3.55, 95% CI 2.31-5.47; *P *≤ 0.001). Concerning race, non-Hispanic Black individuals had a 29% higher rate of ED visits compared to non-Hispanic White individuals (OR = 1.29, 95% CI 1.05-1.58; *P *= 0.016). No other significant differences were seen among the other races or geographic regions.

**Table 3:** Unadjusted and adjusted associations between COVID-19 and suicides. Analysis was performed by using chi-square analysis, *P *< 0.05. Uni- and multivariable logistic regressions to estimate the odds ratio (OR) and 95% confidence intervals (CIs).

Characteristics	Unadjusted		Adjusted		Statistical test
	OR (95% CI)	*P*-value	OR (95% CI)	*P*-value	
Time					
Pre-COVID-19 (2018-2019)	Reference		Reference		
During-COVID-19 (2020-2021)	0.98 (0.84-1.16)	0.847	0.98 (0.84-1.15)	0.844	OR: *P *< 0.05
Sex					
Female	Reference		Reference		
Male	1.46 (1.24-1.7)	0.000	1.53 (1.30-1.81)	<0.001	OR: *P *< 0.05
Age					
18-44	7.33 (5.01-10.73)	0.000	7.24 (4.92-10.67)	<0.001	OR: *P *< 0.05
45-64	3.66 (2.39-5.63)	0.000	3.55 (2.31-5.47)	<0.001	OR: *P *< 0.05
65+	Reference		Reference		
Region					
Northeast	Reference		Reference	Reference	
Midwest	1.09 (0.829-1.44)	0.522	1. 07 (0.817-1.39)	0.627	OR: *P *< 0.05
South	0.818 (0.625-1.07)	0.145	0.80 (0.629-1.03)	0.079	OR: *P *< 0.05
West	0.98 (0.77 - 1.24)	0.874	1.01 (0.805-1.27)	0.935	OR: *P *< 0.05
RACE					
Non-Hispanic White	Reference		Reference		
Non-Hispanic Black	1.48 (1.22-1.81)	0.000	1.29 (1.05-1.58)	0.016	OR: *P *< 0.05
Hispanic	1.22 (0.97-1.53)	0.083	0.978 (0.77-1.24)	0.850	OR: *P *< 0.05
Non-Hispanic Others	0.99 (0.715-0.026)	0.000	0.89 (0.65-1.23)	0.487	OR: *P *< 0.05

## Discussion

Since the onset of the COVID-19 pandemic, concerns emerged regarding its potential impact on mental health, including an anticipated rise in suicidal behaviors, partly due to various stressors associated with the pandemic. These stressors included financial difficulties, isolation resulting from COVID-19 protocols, and concerns about physical health [15]. Based on these factors, we hypothesized that the rate of presentations to the ED secondary to suicide/intentional injury would increase in the US population. Yet, our results indicate that there was no significant change in suicide-related visits to the ED when comparing pre-COVID-19 to during-COVID-19 times. 

Our findings contrast with those of a retrospective study done in Castile and León in Spain, which included 529 participants and assessed the impact of COVID-19 on ED admissions for self-harm [16]. The study reported differences in the number of admissions for self-harm between two periods: pre-pandemic (2018 and 2019) and during the second wave of the pandemic (2021 and 2022), with numbers increasing from 185 to 262 cases admitted for self-harm (185 vs. 262). 

Similarly, another study was conducted using hospital records of 4480 patients from Daegu Metropolitan City in Korea. This study covered one year before and after the COVID-19 pandemic. It revealed a significant increase in the daily mean Emergency Medical Services (EMS) requests related to self-harm or suicide (5.8 ± 2.6 vs. 6.4 ± 2.9). This reflected an overall increase of approximately 10.3% of admissions related to suicide and self-harm during the pandemic versus the pre-pandemic period (n = 4,480; *P*-value = 0.005) [15]. 

Potential reasons for the differences in results may be due to differences in the study location and population makeup. While the study in Spain by Fernandez-Martinez et al. included a smaller sample size (524 individuals) compared to our nationwide study in the United States (1,375 cases), suicide outcomes were assessed within three years following the pandemic. Additionally, Fernandez-Martinez et al. discussed that many of those who attempted suicide had a history of anxiety and/or depression, were on multiple medications, and had previous psychiatric hospitalizations [16]. Zhang et al. suggested higher rates of mental health concerns in Spain compared to the US population [17]. Thus, it is likely that the study in Spain consisted of a higher risk population, for which the outcome events (suicide-related visits) were more common. This higher prevalence provided greater statistical power to assess differences in suicide-related visits over time. 

Other factors that could have contributed to the differences in study results might relate to the differences in healthcare systems between countries such as Korea, Spain, and the United States. Universal or single-payer healthcare in Korea and Spain may influence the patterns of utilization of emergency care for mental health issues, potentially capturing cases of suicide or suicide attempts that could be overlooked in the United States, where universal coverage is absent. This is supported by a study of 1,369 individuals, which reported that 330 (24.1%) of people in need of medical care in the United States would not seek care due to high costs, and 114 (8.3%) would not seek care due to lack of insurance [18].

Furthermore, the varying degrees of impact experienced by each country due to the pandemic should be considered. The emergence of new variants at different intervals, how the population coped with them, and the responses of public health systems were also significant aspects to note. One facet of this that has been previously explored was the healthcare infrastructure specific to the country of study. However, additional considerations may include the baseline resources available, and how each country chose to allocate their resources. Additionally, we must consider cultural, social, and demographic differences, and how these may have an impact on suicide risk. As previously mentioned, a significant rise in suicide rates among elderly populations in Brazil was reported, possibly due to the higher degree of isolation experienced by this subset of the population [12].

Another study conducted in Canada utilized national suicide mortality rates rather than assessing ED admissions for intentional injury. The study found a decline in suicide deaths during the COVID-19 period, from 10.82 deaths per 100,000 individuals between March 2019 and February 2020 to 7.34 per 100,000 between March 2020 and February 2021 [11]. However, it's important to note that this Canadian study captures the worst consequence of suicidal behavior deaths and provides real incidence rates within a defined population. In contrast, our study focuses on ED visits as a proxy for suicide attempts and intentional injury, which only reflects part of the spectrum of suicidal behavior who survived long enough to visit the ER. As such, our results are not directly comparable to the suicide rate trends seen in Canada. Furthermore, our study design, which belongs to the category of proportional prevalence studies, does not allow us to estimate the actual risk of suicide attempts because we do not have population-based denominators. Instead, we report the proportional prevalence of suicide-related ER visits compared to other causes of ER visits. This methodological difference means that our findings should be interpreted with caution, particularly when comparing them to studies reporting population-based mortality rates. Additionally, it's crucial to acknowledge that while underestimation of suicide-related visits to the ED is likely, we have no evidence that this underestimation changed before compared to after COVID-19. However, given the limitations inherent in proportional prevalence studies, such as the potential for biased results, our study cannot conclusively determine whether the pandemic increased the actual risk of suicide attempts.

Regarding direct comparison with US national data, a study by Garnett et al. utilized the US National Vital Statistics System's multiple cause of death mortality files from 2001 to 2021. Their analysis, focused on suicide rates, revealed a notable rise in the suicide rate among men aged 55 to 74 during the aforementioned period [19].In contrast to that study, our research examined rates of suicide-related ED visits, which included suicide attempts rather than suicide deaths. Our study found that older men were seven times less likely to have an ED visit for suicide/intentional injury compared to younger men in general; however, the difference was not statistically significant when comparing pre- and during COVID-19. Future studies are needed to further understand suicide occurrence based on age, gender, and race change before and after COVID-19. 

Further limitations need to be addressed. The main limitation of our study is the design itself, which compares proportional prevalence ratios of ED visits for intentional injury. While this approach allows us to analyze trends in suicide-related behaviors, it does not provide direct measurements of suicide deaths or accurate counts of actual suicides as recorded on death certificates. Instead, our study relies on ED visits related to suicide attempts, intentional injuries, and poisoning as a proxy for suicidal behavior. This design limits our ability to estimate the actual risk of suicide attempts in the population, as we do not have population-based denominators. We acknowledge that a more robust study design, such as a prevalent case-control study, would be needed to overcome this limitation and provide a clearer understanding of the relationship between the pandemic and suicide risk. While redoing the entire study is not feasible at this stage, we recognize and have acknowledged this crucial limitation in our discussion.

The NHAMCS uses data derived from the ICD codes in administrative billing records to define the intent of injury categories. These codes serve as tools for injury surveillance, encompassing intentional self-harm monitoring. Our study investigated both suicidal behaviors and intentional self-harm, the latter of which comprises purposeful self-harm acts without the intent to die. However, it's worth noting that this database may not conclusively distinguish between suicidal and nonsuicidal intentional self-harm ED visits, which is critical for both surveillance and clinical research [20]. 

Out of 54,996 participants, 5,233 (9.5%) had missing data on intentional injury, which were ultimately excluded from the analysis. About 10% of the records of hospital visits related to an injury/trauma, overdose/poisoning, or adverse effects of medical/surgical treatment lacked information on whether the occurrence was intentional. As a result, those cases were not considered potential intentional injuries and were excluded from the analyses, potentially leading to an underestimation of the frequency of intentional injuries. However, the missing data were uniformly distributed across both the pre- and during-COVID-19 periods, suggesting the possibility of nondifferential misclassification. While this could not be confirmed, it is believed that the missing information did not impact the associations reported. Finally, information on certain individual-level covariates that could influence suicide frequency and serve as potential confounders, such as access to healthcare providers, use of medications, therapy, and social network support, among other factors, was unavailable.

While our study highlights important trends, it also raises crucial questions, particularly about the impact of the COVID-19 pandemic on different subgroups. For example, the odds of suicide-related visits may have changed among high-risk subgroups before and after COVID-19. In males, who appear to have higher odds than females, a detailed analysis could reveal whether there was a noticeable change in the odds pre- versus during-COVID-19, suggesting a modifying effect of the pandemic. These specific relationships were not explored in the current study, but they represent important avenues for future research. Therefore, our findings underscore the need for more comprehensive research to fully understand the COVID-19 pandemic's multifaceted impact on mental health and suicide-related behaviors, particularly in examining whether public health crises like a pandemic could exacerbate documented disparities in suicide risk across gender, race, and age groups.

## Conclusions

This study indicates that the proportion of ED visits for intentional injury remained relatively stable before and after the COVID-19 pandemic, suggesting no significant alteration in this particular metric. However, this research uncovered an important incidental finding: increased proportions of suicide-related ED visits among specific demographics, including men, younger individuals, and Black populations. This insight underscores the necessity of examining trends within these vulnerable groups to gain a comprehensive understanding of the pandemic's impact on mental health and suicide rates. The increased proportions of ED visits in these demographics suggest that while the general population may not have experienced significant changes in ED visits for intentional injuries, certain groups have been disproportionately affected by the pandemic's stressors. 

These findings highlight the critical need for targeted interventions and support strategies tailored to the specific needs of these vulnerable populations. Implementing mental health programs, increasing access to counseling services, and creating community support networks are essential steps in promoting mental well-being among these groups. Additionally, ongoing monitoring and research are necessary to track the long-term effects of the pandemic on mental health and adjust intervention strategies as needed.

It is important to note, however, that the study has limitations, such as the lack of analysis of further associations within subgroups. Future research should address these limitations by exploring more granular data on race, age, and gender to better understand the specific mental health needs of these populations. Furthermore, additional research is necessary to investigate other potential associations and contributing factors. In conclusion, while overall ED visits for intentional injury remained stable, the increased suicide-related visits in certain demographics reveal critical areas for focused mental health support and intervention, which future studies should continue to explore.
